# Distal lung organoids derived from adult stem cells as novel tools in deciphering mechanisms of lung regeneration, infection, and cancer

**DOI:** 10.1093/stcltm/szaf040

**Published:** 2025-09-11

**Authors:** Mark Bell, Anna D Krasnodembskaya

**Affiliations:** Wellcome-Wolfson Institute for Experimental Medicine, School of Medicine, Dentistry and Biomedical Sciences, Queen’s university of Belfast, Belfast BT97BL, United Kingdom; Wellcome-Wolfson Institute for Experimental Medicine, School of Medicine, Dentistry and Biomedical Sciences, Queen’s university of Belfast, Belfast BT97BL, United Kingdom

**Keywords:** human tissue models, lung organoids, organoids

## Abstract

While lung research has made great strides in understanding lung physiology, lung pathology still presents a major burden to patients and healthcare systems globally. To develop new effective therapeutics to improve lung regeneration, prevent spread of infections, or treat lung cancers, obscured fundamental processes of the lung must be dissected. Current understanding of lung cell cross talk has been limited due to a lack of accessible and representative models. Since the COVID-19 pandemic, many new foundational methodologies for distal organoid formation have been published, which eliminate difficulty in distal organoid longevity and donor cell extraction efficiency. This review describes how recent advances within distal lung organoid technology have been used to investigate lung regeneration, fibrosis, infection trafficking, personalized medicine, and mechanism of chronic lung pathology using donor cells. Additionally, the applicability of distal lung organoids to investigation of the roles of endothelium and previously unknown distal epithelial and mesenchymal cell populations is discussed. Finally, new techniques and methods for tackling current challenges within the field, such as integration of immune cells and vascularization of organoids are highlighted. This overview will therefore illustrate the potential of distal lung organoids to be tissue representative models, which will be crucial for evolving scientific knowledge of lung physiology.

Significance statement Distal lung organoid technology has transformed lung research by providing more representative models for studying lung regeneration, fibrosis, infection trafficking, and chronic lung pathologies. Recent advances in organoid methodologies have improved donor cell extraction and longevity, enabling deeper insights into lung cell interactions. This review highlights the potential of distal lung organoids to advance personalized medicine and uncover roles of previously unexplored cell populations, including endothelium and mesenchyme. Additionally, challenges in integrating immune cells and vascularization are discussed, emphasizing the importance of organoids in advancing lung disease research and developing new therapeutics.

## Introduction

The lungs are the most vascularized organ in the body and combined with their high surface area and permeability, the lungs have great potential for both systematic and local drug delivery.^1^^,^^2^ However, this also makes the lungs vulnerable to exposure of toxic pollutants and pathogens, which can lead to severe acute and chronic respiratory conditions. Development of therapeutics for lung diseases are crucial, given their heavy burden on global healthcare.^3^ This includes chronic obstructive pulmonary disease (COPD), which was the third leading cause of mortality in 2019,^4^ as well as screening and interventions for lung cancer, which represent the leading cause of cancer-related deaths globally.^5^ Furthermore, the lungs are essential in the replication and spread of infectious diseases, which have long-term impacts on healthcare and economic systems,^6^ highlighted particularly during the COVID-19 pandemic. As such, an accurate and fundamental understanding of lung physiology is needed for management, treatment, and prevention of pathology. Despite this, many key functional aspects of the lung, including regeneration, stem cell characterization, fibrosis, and impact of irritants/infectious diseases are still poorly understood, preventing the successful management of lung diseases.

Insights into these mechanistic pathways have been slowed by a lack of accessible lung models, which could accurately represent the human airways. Previous research heavily relied on the use of murine models, which often failed to replicate findings in humans due to key structural and physiological differences between mouse and human lungs. This is particularly true in the distal airways, where a lack of respiratory bronchioles and differing epithelial populations leads to mistranslation of data, most prominently seen in chronic diseases and regeneration pathways.^7^ While human cells can be used in 2D mono/cocultures for preliminary studies, they are reductionist and do not fully recapitulate the complexity of cellular cross talk and polarization that occurs in vivo. As such, research to develop representative human lung models which facilitate discovery and detailing of currently obscured lung pathways is paramount and has led to the rise of lung organoid models.

Organoids have been defined as “self-organised 3D tissue, which are derived from stem cells, that mimic key functional, structural and biological complexity of an organ.”^8^ Organoids can be derived from three main sources: adult stem cells (ASCs), embryonic stem cells (ESCs), and induced pluripotent stem cells (iPSCs). Due to ethical and accessibility issues, ESCs have mainly been replaced with iPSCs. The use of iPSCs from reprogrammed somatic cells has provided a means to study tissue development. Following reprogramming, iPSCs undergo directed differentiation, where the anterior foregut endoderm is induced by stimulating bone morphogenetic protein (BMP) pathways while inhibiting transforming growth factor beta (TGFβ) signaling.^9^ From here, nearly all lung regions or lung cell populations can be recreated, although standardization of methods is required.^10^ This provides long-lived populations of airway regions/cells for prolonged study, as well as providing previously inaccessible insights into lung development, and its aberrant signaling in genetic diseases such as cystic fibrosis.^11^^,^^12^ This has sparked a wide interest in iPSCs technologies, with the full complexity of the topic outlined elsewhere.^13^^,^^14^ Although induced pluripotent stem cells represent a cutting-edge technology that offer new opportunities for lung modeling, several challenges remain. For instance, there is the potential of iPSC-derived organoids to contain immature cell phenotypes,^15^^,^^16^ or display increased mutational burden,^17–19^ which may impact representative functional activity or impact modeling integrity, respectively. Furthermore, validation of how closely iPSC-derived organoids represent mature human airways is crucial, at both the functional and RNA level, given that incorrect characterization of mouse models led to the obscurity of many lung pathways. For instance, iPSC-derived organoids often tend to match fetal human tissue signatures closer than adult tissues,^20^^,^^21^ with further work being needed to clarify if this impacts the ability of iPSC-derived models for studying the mechanisms of chronic diseases such as COPD.

In contrast to iPSC technologies, ASC organoids can be formed using adult stem cells taken from donors, including epithelial stem cell progenitors with or without fibroblasts and lung mesenchymal stem cells (LMSCs), also known as lung resident mesenchymal stromal cells, to model the airways. Lung mesenchymal stem cells are considered early progenitors of mesenchymal cells, with high potential to self-renew, with their dysfunction being linked to chronic lung diseases such as COPD and Idiopathic Pulmonary Fibrosis (IPF).^22^^,^^23^ The types of organoids generated, as well as characterization of epithelial/mesenchymal progenitors, will be discussed in subsequent sections. Unlike iPSC organoids which are coordinated to form airway organoids via multiple step-wise additions of inducers and inhibitors, ASC-derived organoids self-aggregate by relying on cell–cell specific interactions and secretions.^24^ While organoid formation in ASCs does not directly rely on external inducers given at specific time points, it should be noted that specialized media containing numerous signaling molecules, many of which are found in iPSCs-derived organoid media, is used. The molecules are often added to epithelial ASCs for either expansion or differentiation, due to difficulty in maintaining population doubling and representative airway cell populations. As such, it is important to remember that both iPSCs- and ASC-derived organoids should be considered simplified models of the human airways, which will require further validation via sequencing and future in vivo work. A key benefit to using ASC organoids is that ASCs or cell populations can be accessed from human donors who have lung pathologies, particularly for chronic conditions. This means ASCs may contain epigenetic or functional aberrations that contributed or occurred from lung pathology, allowing for insights into disease states and dysfunction. This provides an opportunity to modularize respiratory modeling, where epithelial cells or mesenchymal cells from healthy donors and patients with lung pathologies can be placed into separate organoids and then compared in terms of size, number, and structural anatomy. Furthermore, organoids can be seeded with pathological epithelial cells and control-derived mesenchymal populations or vice versa, to investigate the influence of cell populations on pathways such as regenerative signaling. Suspected underlying pathways of dysfunction can then be studied using drug treatments or pathway modulators, allowing for a high-throughput and disease-relevant model. This does, however, require access to clinically available samples or tissues, which are often limited given the need for extensive surgeries. This issue is amplified for accessing control or “healthy” donors, which may come from lung rejected for transplantation or from unaffected areas of lung during cancer resection surgery. In a similar manner to iPSC-derived organoids, the influence of signaling molecules in media and long-term cell culture’s impact on ASC organoid representability will require further validation through RNA sequencing and functional comparisons. Despite these limitations, both technologies provide a tissue and disease-relevant model to bridge the gap of in vitro and in vivo works.

As such, primary ASCs allow for an in-depth characterization of disease phenotypes, particularly for lung cancers and chronic disease, while in general, iPSCs research focuses on prolonged cell culture for long-term studies, investigation of genetic diseases, and lung development. A comparison of iPSCs and ASCs technologies, including advantages and disadvantages, is provided in Table 1.^17^^,^^18^^,^^21^^,^^25–39^ While iPSC-derived lung organoids have become incredibly complex, many further questions relating to iPSCs representability and characterization remain, as well as open questions regarding standardization of the technology. The impact of donor cell, reprogramming methods and inducers used, and the impact this has on representability of the model span the entirety of the iPSCs field and have been covered in great depth by numerous reviews.^40–43^ Furthermore, 2D air–liquid interface (ALI) models and their benefits for lung research have been discussed elsewhere^44^^,^^45^ and are not always suitable for modeling stromal, epithelial, and endothelial compartments in tandem. Hence, this review will focus on ASC technologies, the current methods used, and their applications for understanding lung physiology, with a focus on the novel modeling systems for the distal airways.

**Table 1.** Breakdown of ASC and iPSC strengths, weaknesses, and potential applications in organoid modeling.

Method	Origin	Strengths	Weaknesses	Applications
Induced pluripotent stem cells (iPSC)	Reprogrammed patient somatic cells	Non-invasive for donors Can create ethical developmental models Can assembly complex co-cultures of many cells’ lineages, from a single donor Creation of organoids for studying genetic diseases, some of which may be inaccessible for tissue collection More literature available for vascularization of organoids Can be maintained longer in culture High potential for future of regenerative medicine Easier to create control samples for inaccessible tissues	Impact of initial cell type used/maintenance on disease epigenetic memory unclear^25–28^ Slight transcriptional differences/fetal signatures, such as high sex determining region Y)-box 2 (SOX2+) expression compared to ASC organoids^21^ Have been shown to demonstrate different apoptotic signaling than adult donors during drug screening, suggesting validation to ASC organoids may be required for certain pathways^29^ Field requires standardization of reprogramming methods and donor cell choice for definite and widespread use^30^^,^^31^ in iPSCs, in addition to the protocol used to created iPSC-derived organoids More difficult to modularize/remove specific cell types from organoids than ASCs-derived Precise methodology increases initial risk of failure Potential risk of undifferentiated iPSC/immature progenitors remaining in organoids that could influence representability, adding further validation steps Some reported issues in genetic instability, with the impact this has on modeling capacity for certain assay such as drug screening in apoptotic or cell survival assays unclear^17^^,^^18^^,^^32^	Developmental lung studies Studies of inaccessible genetic diseases Could create multi-­organoid systems for wider study of drug responses^33^^,^^34^
Adult stem cells	Lung tissue resident stem cells	Retain disease/epigenetic memory^35^ Show high representability to lung tissue when forming organoids^36–39^ Relatively lower up-front cost Efficiency of donor extraction/expansion rapidly growing Modular approach for choice of initial donor cells Organoids can form much faster than iPSC based	Often limited to rare lung transplantation/resection surgery, especially for control tissues Lacking methods in vascularization/adding immune components Organoid formation depends on poorly defined fibroblast/epithelial cell populations Complexity of organoids is determined by the ASC harvested, which may prove more difficult for generating distal airways due to accessibility issues Accessibility of control donor tissue is poor Like iPSCs, further standardization of the field is required, particularly for ASCs choice, uniform extraction methods, and clear cell markers for separation	High throughput screening for personalized medicine Study of cell cross talk in chronic lung disease progression Investigation into novel cell types in lung health and disease Can help validate iPSC lung models via comparisons

## History and formation of adult stem cell lung organoids

**Figure 1.**
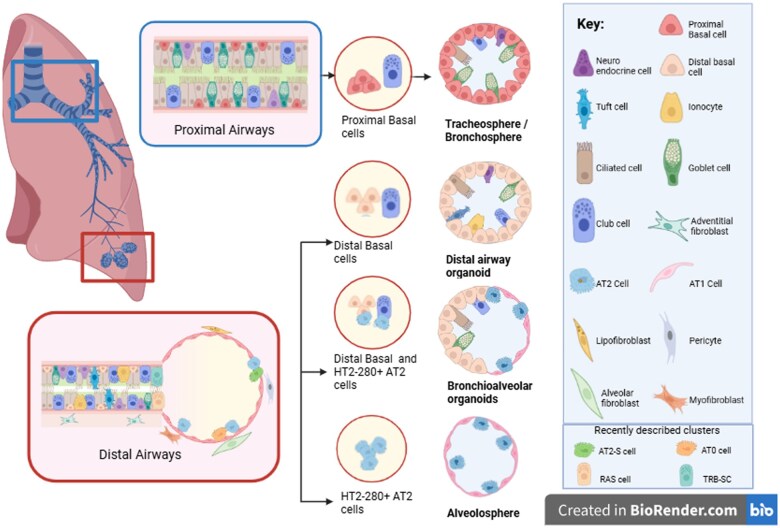
Schematic overview of tracheosphere, bronchospheres, distal airway, bronchioalveolar, and alveolospheres organoids. Epithelium from proximal regions creates tracheosphere and bronchospheres. Tissue digestion of distal airway epithelium can be used to create distal airway organoids or bronchioalveolar organoids,^48^ although a consensus on standardized markers for progenitor basal and club cell populations is needed. Alveolospheres can be created via separation of HTII-280+ AT2 cells as shown in the publication.^49^ AT2: Alveolar type 2 cells, AT1: Alveolar type 1 cells, AT2-S: Alveolar type 2 stem cells, AT0: Alveolar type 0 cell, RAS: respiratory airway secretory cell 80, TRB-SC: terminal respiratory bronchiole secretory cell.^78^ Cell location and subtypes based on cell lung atlases.^36^^,^^37^ Figure created in BioRender.

Generation of lung organoids from lung ASCs can be seen as far back as 1987,^46^ where seeding of mouse fetal lung cells leads to alveolar-like organoids, with mesenchymal maturation into connective tissue. Organoid modeling in larger airways was established in 2009,^47^ where human bronchial tissue and mouse trachea tissue were used to form organoids. These organoids were used to show that keratin 5+ (Krt5^+^), Tumor protein 63+ (TP63^+^) basal cells could self-renew and act as progenitors for other epithelial populations during injury. These organoids are commonly used to model airway regions such as the trachea or bronchioles and are termed “bronchospheres” and “tracheospheres,” while organoids formed from airway epithelial progenitor cells in the distal airways are termed “bronchiolar organoids” and “alveolospheres” if alveolar type 2 (AT2) and alveolar type 1 (AT1) cells are present. This will be reliant on culture conditions and cell origin.^48^ A diagram of identified epithelial progenitors used for airway organoid formation is provided in Figure 1. For upper airway organoids, TP63^+^Krt5^+^ basal cells isolated from the trachea or bronchioles are used, while distal airway organoids are less standardized, often using a mix of cells obtained from digested distal airway tissue pellets, rather than a specific population.^44^^,^^49^^,^^50^ It has been shown that both secretoglobin family 1A member 1 (SCGB1A1^+^) club cells and Krt5^+^ basal cells within the distal airways can form organoids,^51^^,^^52^ suggesting the need for better characterization of the organoid forming populations to enable standardization. Lung organoids can also model the alveoli, using AT2-derived stem cells, which can replace damaged alveoli cells during injury,^53^ and form spherical, self-renewing spheroids in organoid culture. AT2 cells can be obtained via digestion of distal lungs and subsequent isolation of epithelial cell adhesion molecule + (EpCAM^+^), CD31/CD45^−^ cells. The subsequent refinement of AT2 positive cells is often paper specific but commonly involves expanding populations in alveolar differentiation media and segregating AT2 populations using alveolar markers such as human ATII cell membrane antigen (HT2-280^+^) or Lysotracker/SFTPC fluorescence-activated cell sorting (FACS).^39^^,^^54^^,^^55^ A thorough list of proximal and distal lung organoids generated from ASC or iPSCs have recently been described,^44^^,^^56^ with an overview of AT2 organoid formation also available.^57^ Finally, mesenchymal cell populations such as fibroblasts and LMSCs can be extracted from adult lungs and used to support organoid formation. While not essential for airway epithelial organoid formation, inclusion of the mesenchymal niche in organoids allows for modeling of complex cellular cross talk in health and disease. It is important to note, however, that extraction methods used are variable between publications. This can include the scaffold used during explant and if mechanical/enzymatic lung digestion was used, which can influence fibroblast gene expression.^22^^,^^58–60^ Furthermore, due to the complexity involved in identifying various aspects of the mesenchymal niche, these populations will be discussed in the section titled “Organoids for deciphering mechanisms of repair, regeneration and injury in the distal airways.” Nevertheless, ASCs from either the upper or lower airways can be obtained to grow tissue-specific organoids, facilitating complex modeling of the airways.

Often, lung research will focus either on upper lung regions/proximal airways or the distal airways. In general, upper airway contains pseudostratified epithelium, which plays a key role in prevention of infection and removal of pollutants through secretion and removal of mucus by goblet and cilia cells, respectively. The distal airways, which are made up of small bronchioles and alveoli sacs, are fundamental for understanding gas exchange, distal airway regeneration, and cross talk, in addition to studying pathological dysfunction as seen in emphysema or development of adenocarcinoma. The field for modeling larger regions of the airways quickly gained momentum with many bronchial and tracheal organoids being developed, due to easier accessibility of upper airway cells. As upper airway organoid development has progressed, the use of mouse cells has slowly been replaced with human bronchial basal cells. These models have been used to investigate a range of lung physiology and disease, including fibrosis, stem cell characterization in the upper airways, regenerative medicine, chemotherapy efficacy, and more.^35^^,^^61–64^ Despite the success of upper airway organoids, progress in distal airway organoids has been slower. Distal lung organoids often lack region-­specific mesenchymal populations, which are often substituted with Medical Research Council cell strain 5 (MRC-5) fibroblasts or even mouse cells,^44^^,^^65^^,^^66^ with only a few using a mix of human primary small airway epithelial and mesenchymal cells, as will be outlined in the following. This has largely been due to issues acquiring primary distal airway cells in comparison to upper airway cells, as well as difficulty in culturing small airway cells for subsequent passages.

The inaccessibility and high cost of cultures associated with distal airway cells has hindered bronchioalveolar organoid development. As such, many underlying pathologies seen in the distal airways that rely on cross talk between multiple cell types like emphysema, fibrosis, and impaired alveolar regeneration are incompletely understood. However, recent advances in the field are overcoming these obstacles. These include improvements in the long-term 3D culture of small airway cells, more effective lung homogenizing methods, and efficient extraction of donor cells. These breakthroughs have facilitated the start of more complex alveolar organoids, which are of recent discovery. Hence, this review will focus on these novel technologies and their facilitated complex distal airway organoid models, their potential uses, and what issues currently require answering to further research within the field.

## Advances within distal lung organoid models

The characterization of alveolospheres was demonstrated in a landmark publication,^67^ which was the first to use lung organoid models to show AT2 cells were stem cells of the lungs. This highlighted not only that human airway epithelial progenitors (AEPs) could form 3D spheroids but also that cocultured with platelet-derived growth factor receptor alpha (PDGFRα^+^) lung stromal cells (considered to be lipofibroblasts) largely improved organoid growth. This allowed AT2 cells to be proliferated for around a year. Similarly, the prolonged 3D culture of pure alveolar epithelial cells that lack stromal cells were later developed, illustrated in Katsura et al.^54^ for the study of COVID-19 in AT2 cells (with a Star Protocol paper later published^68^). A similar publication also achieved AT2 differentiation, which could successfully be transplanted and differentiated into AT1 cells in a mice injury models.^69^ These cultures replace stromal cells via supplementation with inducers and inhibitors, which is highly appealing for regenerative medicine. The optimization of prolonged feeder-free AT2 cell culture without stromal cells would significantly mitigate off target effects if used in transplantation, resulting in improved scalability by alleviating the need to separate and process cells.^70^^,^^71^ While this finding has higher impact for translational medicine, the ability to form AT2 organoids without a stromal compartment could be highly useful for increasing accessibility of AT2 organoids, or for a focused comparison of AT2 epithelium in control and disease states. These organoids are therefore ideal for high throughput modeling of AT2 signaling, as well as providing a future opportunity in transplantation medicine

Distal airway modeling was further enhanced in Sachs et al.,^50^ which focuses on extended culture of airway organoids from donor tissue, rather than solely AT2 cells. Organoids were composed of basal, club, multi-ciliated, and secretory cells, with an absence of mesenchymal marker Homeobox protein Hox-A5 (HOX5A) and gene signatures of alveolar cells, suggesting effective isolation of distal airway cells. In addition to increasing expansion of multiple populations of epithelial cells, this paper also described the formation of non-small cell lung cancer (NSCLC) tumoroids and airway organoids from cystic fibrosis patient tissue. Organoids were then assessed functionally with novel assays, including respiratory syncytial virus (RSV) infection, forskolin-induced swelling assays, and xenotransplantation. The outlined papers above have since become foundational methodologies within the field, due to their combination of increased cell expansion, donor cell extraction, and array of practical assays to utilize organoid potential. This extended culture growth removes many hurdles associated with 2D distal cell cultures, which lose cell phenotypes and expansion potential at low passages. Not only does this reduce the cost of working with cell types for research but also ensures a more ethical, efficient, and accessible use of donor tissue and its expansion for research and translation. As such, the potential of distal organoids has been noticed, with the field beginning to use alveolospheres and bronchoalveolar organoids to uncover previously obscured pathways, including those in infection, tumorigeneses, and most prominently, regeneration.^72–75^

## Organoids for deciphering mechanisms of repair, regeneration, and injury in the distal airways

Most lung diseases are underpinned by aberrant signaling affecting regeneration, a fact outlined by the increased risk of lung pathology in older patients where stem cell potential is lower.^76^^,^^77^ While a general overview of pathways such as WNT/B-catenin are understood, targeting of these pathways will require subtle understanding of signaling cues, gene regulation, and cell cross talk. This research has been largely hindered due to impaired understanding of each distal cell population, such as LMSCs, fibroblast/stromal cells, AT2, and club cell subsets, and the role they play in distal airway repair. For the alveoli, AT2 cells were shown to be stem cells, which can replenish AT1 cells during homeostasis and injury.^67^ It is accepted that at least 2 main clusters of AT2 cells are observed when RNA sequencing is performed, labeled AT2 and AT2-S, although more recent investigations have challenged this.^78^^,^^79^ Using single-cell RNA sequencing, Travaglini et al.^36^ demonstrated that a majority of AT2 cells exist in a quiescent state that actively repress WNT signaling and produce surfactant proteins to prevent alveolar collapse.^80^^,^^81^ These cells play a role in homeostasis of the lung and have been found to be heavily altered in many lung diseases.^82^^,^^83^ On the other hand, AT2-S cells are characterized as tissue stem cells, sharing similarities to Axin2+ AT2 cells in mice, and can renew both AT2 and AT1 cell populations, as well as regenerating the alveoli during injury.^84^ Beyond these traditional subtypes, a recent publication by Kadur et al.^85^ identified a transitionary AT2 state termed AT0 cells using Katsura et al.’s^54^ method for alveolospheres formation. The identification of transitional AT0 cell state was reinforced by a publication from the human cell atlas project.^37^ AT0 clusters were characterized by high gene expression of AT2 marker surfactant protein C (SFTPC) expression, club cell marker SCGB3A2, and a lack of marker SCGB1A1, which is present in mouse bronchioalveolar stem cells. Kadur showed that following depletion of epidermal growth factor (EGF) and CHIR99021, a WNT agonist,^86^ AT0 cells differentiated into AT1 cells or secretory cells termed terminal respiratory bronchial secretory cells (TRB-SCs). These cells formed in cystic-like sacks seen during organoid restructuring, something unlikely to be possible in conventional 2D culture. AT0 cells were found near alveolar sacks or in the alveoli during injury in primate lungs and therefore are suspected to play a key role in regeneration.

While this finding provides a strong hypothesis that AT2 cells may replenish the alveoli and populations of the distal airways, another study contradicts these findings.^87^ This paper used similar alveolar organoids derived from a specific subset of distal epithelial cells termed respiratory airway secretory (RAS) cells, which underwent unidirectional differentiation in AT2 cells during WNT depletion. The RAS cells also show high SFTPC and SCG3BA2 markers, like AT0. However, AT2 cells derived from RAS cells were not able to differentiate back into AT0- or TRB-SC-like cells when media was reverted, therefore providing contrasting data. Interestingly, both papers showed similar anatomical locations for cells and altered populations in injury and COPD, suggesting that understanding AT0 and RAS cells will be critical in deciphering mechanisms of distal lung repair. The difference in results may be due to non-standardization of organoid generation methods, which is highly likely to occur given the novelty of the technology. For instance, the differentiation media used in Basil et al.^87^ does not remove EGF and contains components such as recombinant human keratinocyte growth factor (KGF), 3-isobutyl-1-methylxanthine, and 2 mM “TZV,” which are not stated in Kadur et al.^85^ This is a common issue within the field, as demonstrated by comparing components used in seminal organoid development papers,^50^^,^^54^^,^^69^ as provided in Table 2. Furthermore, RAS cells were identified with Carcinoembryonic antigen-related cell adhesion molecule 6+(CEACAM6^+^), HT2-280^−^, and nerve growth factor receptor− (NGFR^−^).^87^ Meanwhile, TRB-SCs were mainly identified by SFTPC and SCGB3A2, which may cause poor comparison of cell populations. As the field continues to expand, it will be important to create a clear understanding of what components are added to organoid media, what distinct markers can be used to identify the observed cell type, and how cell culture conditions may affect the representative ability of distal lung modeling.

**Table 2.** Breakdown of media components used in seminal methodologies for distal lung organoid generation.^50^^,^^54^^,^^69^

		Component concentration			
		Katsura et al.^54^ (Feeder Free)	Sachs et al.^50^	Weiner et al.^69^	Expected purpose
Base media	Dulbecco’s modified Eagle medium/nutrient mixture F-12 (DMEM-F/12)	×	×		Base media/nutrients
	Small airway growth media (SAGM)			×	Base media/nutrients
Components	SB431542	10 µM			Blocking transforming growth factor β (TGFβ) signaling
	CHIR99021	3 µM			Glycogen synthase kinase 3 (GSK3)/Wingless Int-1(WNT) pathway activator
	BIRB796	1 µM			Blocking P38 mitogen-activated protein kinase (MAPK) signaling
	Y-27631	10 µM	5 µM	5uM	Blocking Rho-associated coiled-coil containing protein kinase (ROCK) signaling
	SB202190		500 µM		Blocking p38 MAPK signaling
	A83-01		500 µM	1 µM	Blocking of TGFβ signaling
Recombinant proteins	Epidermal growth factor (EGF)	50 ng/mL		50 ng/mL	Epidermal growth factor receptor (EGFR) signaling
	Fibroblast growth Factor (FGF) 10	10 ng/mL	100 ng/mL	100 ng/mL	Fibroblast growth factor receptor (FGFR)2b signaling
	Interleukin -1β (IL-1β)(First 4 days)	10 ng/mL			Increased alveolosphere size
	Noggin		100 ng/mL	100 ng/mL	Blocking of TGFβ signaling
	FGF 7		25 ng/mL		FGFR2b signaling
	Wnt3a			100 ng/mL	WNT/β-catenin signaling
	Keratinocyte growth factor (KGF)			20 ng/mL	Assumed protective epithelial signaling (Portnoy, Curran-Everett and Mason, 2004)^96^
	R-Spondin 1		500 ng/mL	200 ng/mL	WNT/β-catenin signaling
Supplements	Heparin	5 µg/mL			Not stated
	B-27 supplement	1×	1×	1×	Insulin signaling
	Antibiotic-antimycotic	×	×	×	Prevent contamination
	4-(2-hydroxyethyl)piperazine-1-ethanesulfonic acid (HEPES)	15 mM	10 mM	10 mM	Buffer
	GlutaMax	1×	1×		Nutrient
	*N*-Acetyl-L-cysteine	1.25 mM	1.25 mM		Antioxidant
	Nicotinamide		5 mM		Co-enzyme precursor
	N-2 supplement			1×	Nutrient

To understand the precise role of these inducers, as well as validate the role of RAS and AT0 cells within regeneration of the distal airways, more complex organoids containing mesenchymal niches will need to be included. Earlier lung organoids used human MRC-5 or mouse lung fibroblasts (Mlg) when modeling cross talk for a range of pathways, including tumorgenicity, cell proliferation, and regeneration via WNT signaling.^88–90^ These larger and more complex organoids form airway lumens, generally last longer in culture, and can be modulated to include diseased mesenchymal cells to understand regeneration pathways in health and disease. While fibroblastic cell line (MRC-5) provides a much more accessible cell type for co-culture organoids than primary donors, they may significantly reduce representability and translatability of the research. Distal airway organoids will need to contain the correct fibroblast subsets and populations, to ensure tissue specific representation. The diversity of lung fibroblasts established by Travaglini et al.^36^ was novel, and researchers are still attempting to characterize a differences between each subset. These eight subsets of the mesenchymal niche identified were smooth muscle, myofibroblasts, fibromyocytes, adventitial fibroblasts, alveolar fibroblasts, lipofibroblasts, pericytes, and mesothelial, with fibromyocytes and alveolar fibroblasts both being newly described. These subsets are still often mislabeled or interchanged in the literature and lack clear cell markers for effective separation and study. Interestingly, LMSCs were not annotated, demonstrating the demand for more effective characterization for fully deciphering lung research. Within the alveoli alone, populations of myofibroblasts, alveolar fibroblasts, lipofibroblasts, and pericytes are present, suggesting that mechanisms of alveoli repair and homeostasis are intricate and highly controlled. Given their high throughput, AT2 or airway epithelial organoids could use different populations of fibroblasts to compare the impact of each subset on regeneration and activity on epithelial cells, thus allowing characterization of fibroblast populations. Furthermore, cells can be taken from patients with lung pathologies, such as COPD or IPF to contrast between healthy and pathological signaling pathways.

To integrate differing primary fibroblast populations into organoids, reliable separation of each population is needed, as well as defining the role of LMSCs within this niche. Currently, most distal lung fibroblasts are isolated via explant culture, which relies on migration of fibroblast populations rather than cell sorting. Identification of efficient cell markers by which to separate cells is beginning, although mostly in mice, with adventitial and alveolar fibroblasts being reliably sorted with CD34+ and Nephronectin+(NPNT^+^)/CD9^−^, respectively.^91^^,^^92^ CD34^+^ has been used for the separation of human adventitial fibroblasts, which appear to be a possible source of clonogenic mesenchymal cells.^93^^,^^94^ This would suggest that LMSCs are part of the adventitial fibroblast niche, which was corroborated with RNA sequencing data.^95^ In contrast, RNA sequencing performed on LMSCs, BM-MSCs, and AD-MSCs from control and COPD patients found that LMSCs more closely resembled myofibroblasts.^97^ This suggest that fibroblasts or LMSCs extracted from donors can be separated successfully into subsets when correctly characterized, while also demonstrating the importance of validating new extraction methods used, to prevent research miscommunications and allow for representative lung pathway modeling.

The ability to distinguish/segregate lung fibroblast populations, in addition to LMSCs presenting a myofibroblast phenotype could prove invaluable for the study of lung fibrosis. During injury, cells of the mesenchymal niche adapt a myofibroblast phenotype that become contractile and begin to remodel the extracellular matrix to initiate wound healing.^98^ Pathogenic fibrosis can be both the driving force of disease progression as seen in IPF, as well as contributing factor as seen in COPD and asthma.^99^ Organoids for the study of IPF have mostly been iPSC based,^100^ but some papers have already shown the advantages of using primary human cells for modeling. For instance, organoids containing epithelial and stromal cell populations from IPF patients have been generated, ^101^ as well as combinations of LMSCs with epithelial cells from COPD donors.^102^ IPF derived organoids grew larger but fewer organoids, suggesting intrinsic differences in epithelial and stromal cell populations, with a similar trend noted for COPD-derived LMSC organoids. Further experimentation into the epigenetic and genetic differences between control and disease isolated fibroblasts and their subsequent role in organoid supporting capacity could provide essential information for targeting pathogenic processes. Similarly, distal organoids created using AT2 cells with primary fibroblasts with a fibrotic phenotype showed decreased SFTPC secretions while increasing Mucin 5B (MUC5B) expression.^103^ Interestingly, this observation was not seen in MRC-5 fibroblasts, demonstrating the impact of using primary fibroblasts for functional studies. Using pathway inhibitors and anti-cancer drug Dasatinib, this change was shown to be driven by interleukin 6 (IL-6)–STAT3 signaling, which could be partially reversed. This highlights the potential to recycle old therapeutics such as pathway inhibitors for novel disease settings, providing an incredibly cost effective and efficient method for translating therapeutics to the clinic. While use of primary fibroblasts and epithelial cells begins to increase, it is also important to consider the role of the endothelium within fibrosis and repair, as representative models are lacking.^104^

The endothelium is mainly associated with providing nutrients and waste disposal for tissues, but it also regulates many homeostatic functions, including regeneration, fibrosis, and inflammation. A full overview of the endothelium in lung homeostasis and disease has been described previously.^105^^,^^106^ For distal airways, the endothelium has been shown to influence epithelial cell proliferation via secretion of hepatocyte growth factor (HGF) and epithelial growth factor (EGF), modulating the masking of EGF receptors to increase AT2 proliferation,^107^^,^^108^ direct epithelial cell differentiation,^109^ and regulate parts of the inflammatory response.^110^ Given their role in regeneration and fibrosis amelioration,^111^^,^^112^ it is not surprising that loss of endothelial cell function is associated with lung pathology. For instance, loss of endothelial markers vascular endothelial growth factor A (VEGF-A) and VEGFR2 in COPD is directly correlated to increase airflow obstruction, while restoration of the endothelium can ameliorate alveolar destruction in emphysema.^113^^,^^114^ Despite their incredible potential to facilitate regeneration in a healthy state, dysfunctional endothelium can actively reduce lung regeneration potential. This is seen during frequent injuries to the endothelium with bleomycin, which caused the downregulation of C-X-C chemokine receptor 7 (CXCR7), leading to increased fibrosis via Notch-­dependent fibroblast activation.^115^ Similar studies have shown that dysregulation of endothelial cells, either through chromatin remodeling in aging or altered glycolysis, could prevent fibrosis resolution via abnormal paracrine signaling.^116^^,^^117^ To study how the endothelium directs fibroblasts to a pro-regenerative/fibrotic state in health and disease, the complex underlying cross talk between polarized cells needs to be modeled.

While an established protocol for studying endothelial,­ ­epithelial and mesenchymal cross talk in the distal airways is lacking, a proximal lung organoid containing primary human bronchial epithelial cells, primary human fibroblasts, and human pulmonary microvascular endothelial cells (HPMECs) was used to study fibrosis.^118^ By modifying these protocols with SAECs and alveolar fibroblasts, a distal lung organoid could be created to study cross talk signaling. Furthermore, a recent paper used general and aerocyte capillaries’ cells to form branching vasculature, which increased organoid lifespan,^45^ while another publication has created cell line suspension organoids to model vessel formation at the alveoli and the impact of bleomycin on fibrosis.^119^ As well as providing insight into distal airway regeneration that was not previously possible for human models, it will be highly interesting to study if healthy endothelium can reverse or ameliorate dysfunctional signaling seen in donor cells from diseases like COPD and IPF, providing a strong basis for further transplantation medicine. Addition of endothelium also provides an opportunity to vascularize the organoid, which is essential given the lung is the most vascularized organ in the body. In previously mentioned papers,^45^^,^^118^ it was shown that organoids with endothelial cells were larger and longer lived, although true vascularization is unlikely to be present. Regardless, the presence of the endothelium likely increased perfusion via formation of simple endothelial vessels that attenuate development of a necrotic core or was crucial in proliferation and communication of epithelial cells and the mesenchymal niche. The topic of vascularizations spans the breadth of the organoid field, with many current challenges including endothelial fragility, optimizing extracellular matrix (ECM) stiffness, and implementing mechanical flow.^120^^,^^121^ Defining when an organoid is truly vascularized, rather than containing endothelial cells, also needs to be standardized, but research is promising. Studies within the adult stem cell field have already been successful for liver and kidney ASC organoids,^122^^,^^123^ and organoids vascularized in the presence of mechanical flow^124–126^ have been developed. The presence of fluid flow would allow for the study of mechanical and hydrostatic forces on endothelial controlled regeneration,^127–129^ which is imperative for understanding of lung homeostasis and pathophysiology of fibrosis and emphysema. While no ASC lung organoids have currently been formed in the presence of microfluidic flow, its development will aid in fundamentally understanding endothelial and distal airway biology.

While much work is still required, the development of distal lung organoids for distal airways and alveoli provides a gateway for deeper understanding of regeneration, repair, and fibrosis. By using patient donor cells, disease models that recapitulate human disease can be created providing new avenues to illicit exact pathways underlying these processes, as well as defining the role of the mesenchyme, endothelium, and epithelium. Furthermore, progression in extended culture methods and better-defined cell subtypes will allow more accessible and standardized data, that should lead to easier translation of therapeutics targeting lung regeneration.

## Investigating infectious diseases in the distal airways

To gain a full insight into pathogen biology, it is essential that their infection and replication of host cells are understood. The 3D lung organoids display cell polarity.^38^ For instance, organoids have been shown to display correct cell orientation with basal, goblet, and ciliated cells on the lumen facing side, as seen in vivo,^130^ using methodology based off Sach’s et al.^50^ Therefore, lung organoids provide the opportunity to investigate proteins where polarization is needed, such as IL-17,^131^ or virulence factors for infection^132^ due to their organized and representative structure. Furthermore, organoid polarity or site of infection via apical/basal injection can be modified, to provide a much deeper understanding of entry mechanism and infection impact on barrier integrity compared to 2D culture.^133^^,^^134^ Organoids are therefore highly appealing to study infection, although a majority of research has focused on upper airway models rather than distal, due to previously mentioned accessibility issues.

While distal lung infection modeling was initially sparse, research focusing on viral infection of Influenza^135^ and COVID-19 has rapidly increased since the pandemic. This increase is partially due to previously mentioned seminal papers published by Sachs et al.^50^ and Katsura et al.,^54^ who included viral infection assays to display dynamics of organoid culture. This included infecting organoids with RSV to cause cytoskeletal rearrangements and increase motility of organoids that increased with NS2 protein overexpression, and utilization of alveolospheres to demonstrate the role of interferons (IFNs) in COVID-19 infection, validating that low IFN dosage could reduce viral titers. The underlying mechanisms of type I/III interferon response programs in AT2 cells and club cells was detailed later using bronchoalveolar organoids,^136^ which provided a mechanistic explanation for trends seen in clinical trials.^137^ Bacterial infection of lung organoids has also been described, including *Mycobacterium abscessus* or *Pseudomonas aeruginosa*, although much of this work occurs in proximal bronchial airways rather than distal.^75^^,^^138^ Viral and bacterial infection of lung organoids have demonstrated functional impact of small molecule inhibitors or pathway activators, including NAD(P)H dehydrogenase quinone 1 (NQO1) with antibiotic cefoxitin or anti-viral Remdesivir to understand mechanisms or drug efficacy.^75^^,^^139^^,^^140^

The most impactful papers for distal lung organoids would combine mechanistic and screening aspect with primary donors with lung pathology, particularly for infections relevant to exasperations or disease. For instance, lung tumor organoids from untreated NSCLC patients were generated and had increased infection susceptibility to influenza.^141^ Furthermore, these organoids displayed adenocarcinoma identity marker thyroid transcription factor-1 (TTF-1), and unlike 2D cultures, these organoids displayed nuclear localization of *M* gene. Another example was demonstrated in Chan et al.,^35^ using bronchial and nasopharyngeal cells to create COPD epithelial organoids. These organoids could be made apical or basal outward facing and were infected with SARs-COV-2 following transcriptomic analysis. The COPD organoids showed lower cilia beat frequency and higher MUC5AC secretion, in addition to higher viral replication, representative of COPD patients.^142^

The functional assays highlighted could be implemented with previously described distal lung methodology, to allow study of infection in disease-relevant models of exacerbations. For example, COPD or cystic fibrosis distal organoids could be used to study the most common pathogens linked to exacerbations such as nontypeable *Haemophilus influenzae*, *Streptococcus pneumoniae*, or *Staphylococcus aureus*.^143–145^ Differences in inflammatory signaling between control and pathological derived distal organoids or the longer-term impacts on regenerative potential of airway cells could be investigated. Conceptualizing how COPD, cystic fibrosis, or other complex lung pathologies signal during infection and inflammation would be novel, as these diseases are poorly modeled in mice. In the case of COPD, cigarette-induced COPD pathology can take 3-6 months and may lack key features including small airway/vascular remodeling, impaired lung function, and infectious exasperation,^146^^,^^147^ while patient-derived xenografts (PDXs) for lung cancer require surgery of mice. Due to the large number of resources required and ethical issues from long-term suffering for potentially non-translatable data, ideally, organoids will replace a large amount of animal models in preclinical research, for both infection and regeneration studies.

Before this can be realistically achieved, it will be essential for distal lung organoids to sustain or interact with relevant immune cell populations, although this been difficult across the field. Immune cells like macrophages have limited proliferation capacity once differentiated and must be extracted from donors regularly, requiring patient and ethical access. As such, mouse models are often used due to similarities of their innate and adaptive immune system and range of genetically engineered models. Furthermore, iPSC technology can be adapted to create both endo/mesoderm lineages to provide endothelial and immune cell as seen in intestine and liver organoids, making them more appealing for studying immune pathways. The wide range of cell types derived from iPSCs, which all stem from a single donor,^148^^,^^149^ and mouse models with adjustable immune systems, present a drawback to choosing ASC for immune system modeling. Despite this, research within the field has been steady. Researchers have successfully implanted lymphocytes into intestinal organoids, which were able to integrate and migrate throughout the epithelial layer,^150^ suggesting feasibility. Within the field of lung organoids, iPSCs-derived macrophages have been introduced to organoids following *Mycobacterium* infection, with immune cells observed engulfing bacteria at the organoid edges.^130^ Alveolar macrophages have also been extracted from bronchoalveolar lavage (BAL) and co-cultured with bronchoalveolar organoids, although this study was based on mice.^151^ Modification of these processes will likely lead to an achievable methodology for the generation of primary human lung organoids, which may involve co-culture or injection of BAL-derived immune cells like macrophages, although this may cause issues when combining BAL and lung tissue from different donors. While this will require much research, inclusion of immune cells will provide an optimal opportunity to uncover human immune signaling in regeneration, infection, and lung cancer organoid models.

## Lung cancer organoid models

Distal airway cancers include adenocarcinoma and large cell carcinoma and contribute significantly to global cancer deaths worldwide, with adenocarcinoma being the most common lung cancer.^152^ Ideally, lung cancer models would be both highly representative of tumor heterogenicity, while allowing high-throughput screening for drug discovery. Mouse modeling of tumors usually involves mouse PDX model, which are often limited in statistical power due to long study periods and poor success rates.^153^ Hence, PDX models are usually unsuitable for predicting optimal drug choice for patient personalized medicines. While iPSCs appear an attractive alternative, a recent study showed a large discrepancy between apoptotic genes signaling following drug treatment in lung cancer organoids, suggesting the importance of comparing iPSC-derived organoids before pursuing further mechanistic research.^29^ Furthermore, iPSC-derived organoids often require more time to develop than ASC-derived organoids, which present a major drawback if using organoids to predict chemotherapeutic efficacy for patients. Distal lung cancer modeling from patients has become more accessible following a seminal paper in 2019,^154^ which provided a fundamental technology for deriving a range of lung cancers with high success and survivability compared to 2D culture. This also established that cancer models retained genetic modification and markers over several passages, agreeing with previously mentioned findings.^155^ Recently, a real-world study that formed tumor organoids was performed, with 80% success rate.^156^ These organoids reflected pathology and tumor organization as shown by spatial transcriptomics. Influenza infected tumors corroborate these findings,^141^ in that lung cancer organoids can be high throughput and representative of patient tumors.

The ability to generate patient-specific organoids rapidly is essential in the field of precision medicine, where organoids could be used to predict clinical outcome of therapeutics. This was confirmed when organoids generated from patients were treated with tyrosine kinase inhibitors, to assess drug efficacy.^72^ The study showed that tumors generated from resistant patients following 4 months of treatment and patients lacking EGFR mutation had largely different dose–response curves to Gefitinib and Crizotinib, which reflect clinical and PDX responses. Given the short time scale of a week for organoid generation, it would be possible to use tumor biopsies to assess drug treatment efficacy for patients, largely increasing treatment efficacy and survival.

While drug screening is a highly important aspect of in vitro modeling, tumors must also be studied mechanistically, to understand tumor organization for novel therapeutic interventions. A large benefit of using PDX models is the presence of vasculature, allowing for a more complete understanding of tumor microenvironment and vascular cross talk. While many current organoids simply use epithelial cells from cancer patients, primitive lung in dish models have been created, using human umbilical vein endothelial cells (HUVECs), MRC-5 fibroblasts, and NL20 epithelial/cancer epithelium.^61^ This model highly recapitulates metastatic tumor morphology, was responsive to anti-VEGF biological therapeutic bevacizumab, and displayed much higher sensitivity to chemotherapy agent 5-Fluorouracil(5-FU) than 3D tumor spheroids. While this model lacked primary endothelial and fibroblast cells, methods to identify alveolar, adventitial, and myofibroblast populations could be implemented to create a personalized organoid.^157^ This is required as it was demonstrated that myofibroblasts populations were associated with worse patient outcome, while adventitial and alveolar populations correlated with improved patient outcomes.^157^ As with studying regeneration, fibrosis, and infection, a more sophisticated distal organoid could be used to discover new cross talk pathways or lung tissue functions that will be essential in the treatment of lung disease.

## Limitations and future work

While lung distal organoids clearly have potential as an effective in vitro model, it is important to outline limitations in the field for future progress. By design, organoids are not complete replicas of their host tissue and will not capture the full complexity of the distal airways. As such, while their use can significantly reduce reliance on mouse models, they will not fully replace them, or human volunteers in early clinical trials, at least of yet. Organoids will also not replace 2D culture within research, given their higher expense and requirement of primary donor tissue for effective modeling, which will require a strong collaboration between clinical and academic researchers. Furthermore, for fundamental research involving tracking of molecular pathways, organoids lack effective tools, often being more difficult to transfect and manipulate in comparison to simpler 2D cultures. It is therefore expected that organoids will occupy a new niche in research: the discovery and translation of biological mechanisms at the tissue level.

Other limitations include lack of flow-based vascularized models, implementation of immune cells from patient donors, and importantly, difficulties in modeling of ECM/appropriate scaffolding. Currently, ECM is a major limitation within the organoid field due to the heavy reliance on Matrigel, which is derived from mouse Engelbreth–Holm–Swarm mouse sarcoma. While more accessible for labs without facilities for hydrogel creation, Matrigel is highly variable and poorly characterized, limiting organoid potential for translational medicine.^158^ Due to being non-tissue specific and murine, it is also possible it may impact signaling of pulmonary cells, heavily altering the biomechanics and structural orchestration in comparison to human ECM. The role of designing appropriate scaffolds for lung organoids, which can support regional specific growth of the airway, is highly complex and has been reviewed in depth in previous studies.^159–161^ Work has already begun to create tissue-specific ECM, such as specialized hydrogels with defined composition/modifiable stiffness or ECM obtained from healthy or pathological homogenized lungs.^162^^,^^163^ These ECMs would allow the study of biomechanical forces and alterations of ECM in disease and improve organoid representability, with ECM research quickly increasing in momentum.^164–167^ It should also be noted that the novelty of the organoid research will lead to difficulties in standardization, especially when additional inducers or variation of extracellular components are included without validating their impact on representability. Standardization of the technology will require prolific scientific discussion but must occur to prevent the escalation of data incompatibilities.

Future work in the field will continue to innovate distal lung organoids, so they are optimal for mechanistic and translation research, thus providing a solution to previous modeling issues. Distal lung organoids will be key in exploring the role of fibroblasts, club cell, and AT2 subsets in regeneration. By uncovering the fundamental science of these stem-like cells, research can answer many questions regarding mechanisms of lung repair, which is the cornerstone of lung homeostasis and disease. Implementing patient-specific cells into high-throughput organoids will also open the door for precision medicine in lung cancer, and possibly fibrosis. Like the rest of the organoid field, the seeding of immune and endothelial cells into distal lung organoids will be needed to significantly advance representative ability of infection, cancer, and regeneration, while balancing the resources required to achieve this. Ultimately, the importance of organoid research is best reflected by its growing use, applications, and potential. As a new representative model for pre-clinical mechanistic and therapeutic screening, distal lung organoids will be a crucial step toward reducing mortality and burden of global lung diseases.
